# Effect of Microcrystalline Cellulose on the Properties of PBAT/Thermoplastic Starch Biodegradable Film with Chain Extender

**DOI:** 10.3390/polym14214517

**Published:** 2022-10-25

**Authors:** Haitao Lang, Xianlei Chen, Jiarong Tian, Jing Chen, Mengna Zhou, Fangfang Lu, Shaoping Qian

**Affiliations:** 1 School of Materials Science and Chemical Engineering, Ningbo University, Ningbo 315211, China; 2 Zhoushan Institute of Calibration and Testing for Quality and Technology Supervision, Zhoushan 316000, China

**Keywords:** PBAT, starch, microcrystalline cellulose, composite, biodegradable

## Abstract

Poly (butylene adipate-co-terephthalate) (PBAT) is a fully biodegradable polymer with toughness and ductility. It is usually compounded with thermoplastic starch (TPS) to balance the cost for manufacturing biodegradable films such as disposable plastic bags. However, blending with TPS reduces valuable tensile strength, which limits the bearing capacity of PBAT film. In this study, microcrystalline cellulose (MCC) was employed as a reinforcement to strengthen the PBAT/TPS biodegradable film. The effect of MCC content on the mechanical, thermal, and morphological properties of the composite film were investigated. The optimal tensile strength and elongation at break reached 5.08 MPa and 230% when 4% MCC was added. The thermal stability and thermal resistance were improved with the addition of MCC; for example, T_max_ increased by 1 °C and T_onset_ increased by 2–8 °C. Moreover, good compatibility among PBAT, TPS, and MCC can be achieved when the MCC content was below 6%. Consequently, the optimal MCC content was found to be 4%. These results could provide experimental data and method support for preparing high-performance PBAT hybrid films.

## 1. Introduction

Poly (butylene adipate-co-terephthalate) (PBAT) is a copolyester of polybutylene adipate and polybutylene terephthalate, and its mechanical properties are similar to low-density polyethylene. PBAT is the most popular polymer that can be used for biodegradable plastic film bags due to its high elongation, excellent toughness, and good heat resistance [1]. However, high cost and poor tensile strength become obstacles, limiting its wide application. As a consequence, many kinds of fillers have been employed to improve the properties of the PBAT composites, including silane [2], lignin [3], nanoclay [4], calcium carbonate [5], fermented soymeals [6], etc. The tensile strength of the hybrid film can be increased by 30–60%, making it technically comparable to polyethylene (PE) film. Nonetheless, due to the limitation of filler content (usually less than 30% to avoid the excessive degradation of certain performances [7]), the issue of high cost still exists [8]. Strategies for synchronously reducing cost and maintaining the biodegradability of PBAT remain to be further investigated. 

Starch is considered as an attractive biodegradable filler for PBAT to cut down the cost. Due to the hydrogen bonding inter- and intra-molecules, natural starch molecules crystallize into microparticles, making it agglomerate and difficult to homodisperse in a PBAT matrix. Consequently, plasticizers (such as water and glycerin [9]) are often employed to prepare thermoplastic starch (TPS), which is much easier to compound with PBAT. Even so, the weak interfacial compatibility between PBAT and TPS significantly decreases the properties of the composites, especially tensile strength. Thus, the additional amount of starch is usually less than 40% in industrial manufacturing [10]. The extension of a molecule chain and the introduction of high-efficiency reinforcements can improve the mechanical properties of PBAT/TPS composite films, and further increase the TPS additive amount to reduce costs.

Microcrystalline cellulose (MCC) is considered as a desirable reinforcement for improving the mechanical properties of polymer composites because of excellent biodegradability, high strength and low cost [11,12,13,14]. Vincenzo Titone et al. [15] found that MCC could congruously embed in the PBAT matrix with a good adhesion so that the particles tripled the elastic modulus of the composites with 20% MCC. Chen et al. [16] investigated the best increase in the tensile strength of TPS film reinforced with 6% MCC. They found that a strong solid hydrogen bonding network between cellulose and TPS was formed. Reis et al. [17] prepared a PBAT/TPS/MCC composite through a two-step method, and found that MCC with a mass fraction below 5% had no significant enhancement effect. In addition, chain extenders such as maleic anhydride [18], polyvinyl alcohol [19], reactive epoxy compatibilizer (L-335A) [1], and tartaric acid [20] were used to improve the interface property of PBAT composites. The commercially available ADR-4386 is widely used to extend the molecular chain of polyesters to increase the properties of biodegradable composites; for example, it has been proven to improve the elongation at a break from 23.5% to 410.3% of PBAT/polylactic acid composites [21]. However, the effect of ADR-4386 in the PBAT/TPS/MCC system still remains to be investigated. In addition, the reinforcement effect of MCC with high TPS contents (>50%) seems unclear as well. 

In this study, the effects of MCC and chain-extender in the PBAT/TPS composites were investigated. Different contents of MCC were adopted to fabricate PBAT/TPS/MCC composites by melt-blending and the extrusion method, and the commercially available ADR 4368 was used as chain-extending agent. The surface chemical and morphological characteristics were studied by Fourier transform infrared spectroscopy (FT-IR) and scanning electron microscopy (SEM). The mechanical properties, thermal properties, thermal degradation properties, and crystallization properties of the composites were characterized as well. Based on these results, the optimal usage amount of MCC was gained accordingly. The results of this study could provide theoretical guidance for the preparation and practical application of PBAT composite films.

## 2. Materials and Methods

### 2.1. Materials

PBAT (TH801T) was produced by Lanshan Tunhe, Co., Ltd. (Xinjiang, China). The density was 1200–1280 kg/m^3^ (ISO 1183), and the melt flow rate was 2–5 g/10 min (2.16 kg, 190 °C). MCC (Alfa-A17730) was provided by Sinopharm Chemical Reagent, Co., Ltd. (Shanghai, China), and the particle size was Ca. 50 µm. Wheat starch, produced by Ganzhiyuan Sugar, Co., Ltd. (Nanjing, China), was a food-grade ingredient available in local supermarkets. Glycerin and urea, ACS grade, were used as bought (Aladdin, Shanghai, China). The commercial chain-extending agent (Joncryl-ADR-4368) was provided by BASF SE (Ludwigshafen, Germany), and the molecular weight was 6800. All other reagents and solvents were used as received from the commercial source.

### 2.2. Preparation of TPS

According to previous work [22], starch, glycerin, and urea were mixed at a weight ratio of 70:25:5. A mixture of 4 g was fed into a WLG10A co-rotating twin screw extruder (Xinshuo Co., Ltd., Shanghai, China) under 120 °C and 100 rpm. The diameter of the screw was 20 mm and the length–diameter ratio was 27:1. Then, the mixture was blended, extruded, and granulated into TPS particles by hand with a size of Ca. 3 mm.

### 2.3. Preparation of PBAT/TPS/MCC Composites

PBAT, TPS, MCC, and ADR-4368 were weighed in proportions according to Table 1. Then, the mixture was fed into a co-rotating twin screw extruder system (HAAKE PolyLab OS, Karlsruhe, Germany) with a diameter of 20 mm and a length–diameter ratio of 35:1. The temperature zone was set to 120 °C/125 °C/130 °C/140 °C/145 °C/150 °C/150 °C/145 °C/140 °C/125 °C. The rotation speed was set at 100 rpm. After melt-blending, the compounds were extruded to film with a thickness of 0.3–0.5 mm through a cast film die.

In the investigation of the impact of a chain extender (ADR-4386), the experimental group with 2% MCC was selected as a comparison. During the experiment, the performance representatives of this group can fully reflect the role of chain extenders.

### 2.4. Tensile Tests

Tensile tests of PBAT/TPS/MCC composite films were performed by a universal testing machine at room temperature (CMT4503, MTS Inc., Shenzheng, China). The films were cut along the extruding direction into dumb-bell-shaped specimen with a length of 50 mm, cross-sectional width of 4.0 mm, and initial gauge length of 30 mm [23]. Five specimens were tested, and the average value was taken for analysis with a settled tensile crosshead rate of 50 mm/min.

### 2.5. X-ray Diffraction (XRD)

X-ray diffraction patterns of PBAT/TPS/MCC composites were obtained using the Xpert PRO (D8 Advance, Bruker, Karlsruhe, Germany) machine with Cu (k_α_) radiation (λ = 0.15406 nm) operating at room temperature, 50 mA and 40 kV. The scanned 2*θ* region ranged from 5.0° to 60.0° with the rate of 0.01°/s. The relative crystallinity index (*CI*) was calculated by using Equation (1) [24],

(1) $$CI(\%)=\frac{A_{c}}{A_{t}}\times 100=\frac{A_{c}}{A_{c}+A_{a}}\times 100$$

where *A_c_* means the crystalline area, *A_a_* means the amorphous area, and *A_t_* means the total area. 

### 2.6. Scanning Electron Microscopy (SEM)

A field emission scanning electron microscope (S-8010, Hitachi, Tokyo, Japan) was used to observe both the surface and the fractured surface of PBAT/TPS/MCC composites. The fractured surface observation sample was collected after tensile test. Before observation, samples were coated with a golden layer. The emission voltage of the electron microscopy was 4.0 kV.

### 2.7. Fourier Transform Infrared Spectroscopy (FT-IR)

The Thermo Scientific FT-IR (Nicolet6700/870, Nicolet, Rhinelander, WI, USA) was used to examine the surface functional groups of the PBAT/TPS/MCC composites. The scanning wavenumber of 4000–400 cm^−1^ with a 4 cm^−1^ spectral resolution was tested. Before scanning, samples were ground into powder, and compressed with KBr powder into thin slices. Each sample was scanned 64 times.

### 2.8. Thermo-Gravimetric Analysis (TGA)

The thermal stability of the PBAT/TPS/MCC composites was determined by a thermo-gravimetric analyzer (STA 2500 Regulus, Netzsch, Selb, Germany). Approximately 10.00 mg of the sample was weighed in a standard alumina crucible and heated from 30 °C to 600 °C at a heating rate of 10 °C/min under a nitrogen atmosphere. An empty crucible was used as a reference.

### 2.9. Differential Scanning Calorimetry (DSC)

The thermal properties of the composites were tested by DSC (200F3, Netzsch, Selb, Germany). Approximately 10.00 mg samples were heated from room temperature to 180 °C at a rate of 10 °C/min, kept for 3 min, and then cooled down to −60 °C at a rate of 10 °C/min. Subsequently, it was heated again to 180 °C at a rate of 10 °C/min. Nitrogen was used as a protecting and purging gas. The second DSC thermograms were recorded for further evaluation. An empty crucible was used as a reference. The PBAT crystallinity (*X_c_*) was estimated according to the following Equation (2):

(2) $$X_{c}(\%)=\frac{\Delta H_{c}}{\Delta H_{0}\times X_{PBAT}}\times 100$$

where Δ*H_c_* refers to the crystallization enthalpy of composites; Δ*H*_0_ refers to the enthalpy value during 100% crystallization of PBAT, which is 114 J/g [25]; *X_PBAT_* refers to the weight ratio of PBAT in composites.

## 3. Results and Discussion

### 3.1. Mechanical Properties Analysis

**Figure 1:**
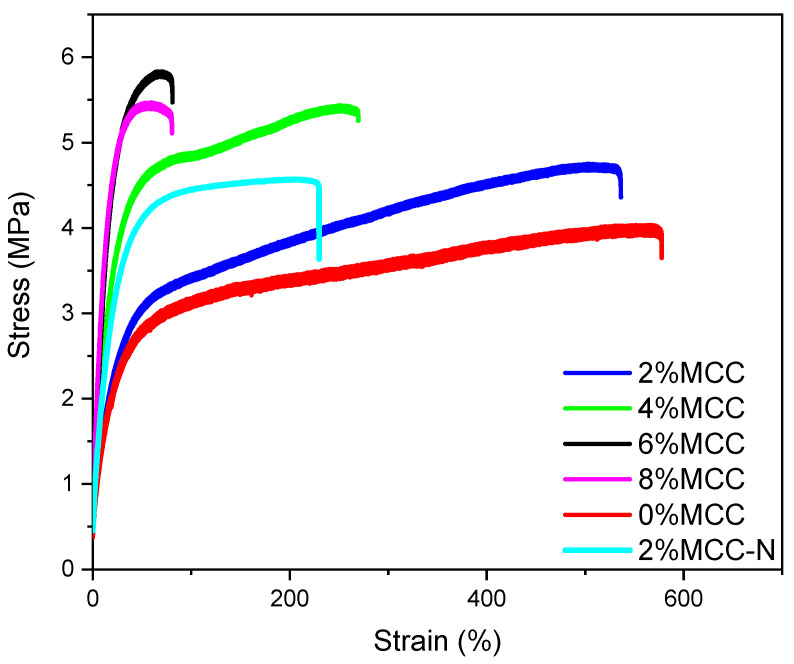
The stress to strain curves of typical film samples with different MCC content.

The tensile properties including tensile strength, Young’s modulus, and elongation at break are tabulated in Table 2, and the typical curves of the stress–strain of different samples are shown in Figure 1. It indicates that the tensile strain tends to be lowered, whereas stress exhibits an enhancement with the increase in MCC content. The tensile strength is enhanced by Ca. 30% (from 4.03 MPa to 5.78 MPa) along with the MCC content increase from 0% to 6%, which obviously suggests that MCC acts as a reinforcement in the composites. Moreover, a steady increase in Young’s modulus is noticed as well. It is remarkably improved by Ca. 200% when compared the film with 6% MCC to that without MCC. This is quite different from the effect of MCC in the polylactic acid matrix, which showed decreases in both tensile strength and modulus [15]. This might be contributed to the interaction of MCC with TPS. As we know, TPS is a ductile material with low modulus, whereas MCC exhibits high stiffness, crystallinity, and modulus [26]. More importantly, they have a similar surface functional group. Thus, MCC and TPS are likely to form hydrogen bonds and mechanical interactions when compounding. These analyses corroborate the results of FTIR and the micromorphology analysis.

Notably, the elongation at break of the samples decreases, especially when the MCC content exceeds 4%. Additionally, when the MCC content is 8%, the elongation at break reaches a minimum. Perhaps the aggregation of MCC decreases the continuity of the different phases of the composites, leading to stress concentrations [27]. Furthermore, in the absence of the chain extender case, the tensile elongation of the composites decreases by Ca. 80%, and the tensile strength remains almost unchanged, indicating that a chain extender mainly plays the role of increasing the ductility. This could be attributed to the fact that the chain extender ADR-4368 improves the compatibility of PBAT and TPS. Wei et al. [10] also found that the chain extender had a cross-linking effect on the PBAT and TPS molecular chains, which also helped tensile elongation. However, it is also worth to consider questioning an efficient approach to premix MCC with the granular PBAT and TPS matrices before melt-blending. The optimal content is found to be 4% after a comprehensive comparison of the tensile strength, modulus, and elongation at break.

### 3.2. XRD Analysis

**Figure 2:**
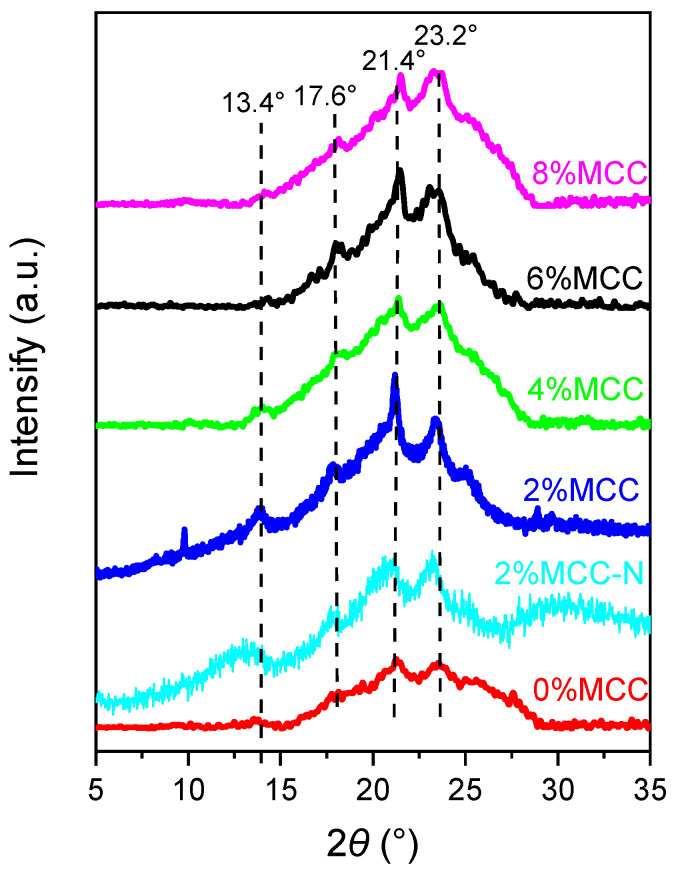
XRD patterns of the samples.

By the X-ray diffraction technique, the results of the crystalline structures of the composites are shown in Figure 2. Four diffraction angles corresponding to the 2*θ* at 13.4°, 17.6°, 21.4°, and 23.2° are observed in the PBAT/TPS/MCC composites. The peaks identified at 17.6° and 23.2° are the characteristic diffraction angles of the PBAT crystalline structure. The diffraction angle of cellulose is supposed to be shown at 2*θ* of 22.5°, which is overlapped by the PBAT crystalline peak [17]. The relative crystallinity index of composites without MCC addition is Ca. 9.7%, which can be increased to Ca. 11% after introducing MCC.

The crystal diffraction peaks of the PBAT/TPS without MCC are very weak and basically present an amorphous dispersion feature. This may be due to the weak crystallization capacity of TPS and PBAT—crystal nuclei can hardly form in the system. Garalde et al. [28] also reported a weak crystal diffraction peak, as the addition of TPS was 40% in the PBAT matrix. With the addition of MCC, PBAT crystallization is facilitated by the heterogeneous nucleation, and the crystalline diffraction peaks at 17.6° and 23.2° are strengthened. This is quite different from the observation of Reis et al. [17]. With the crystallization of PBAT, the tensile strength of the composites gradually increases and the tensile elongations decreases. Interestingly, without the chain extender, the crystal diffraction peaks become small, indicating the weakening of crystallization. Souza et al. [29] also reported an increase in crystallinity once the chain extender was added. It is worth noting that starch itself is a crystalline particle; generally, there are crystalline diffraction peaks at 13.1° and 19.8°. However, in this study, because of the thermoplastic processing, the typical crystalline diffraction peak (2*θ* = 13.4°) for starch is too weak to be seen in the composites. Therefore, it can be deduced that starch has been plasticized during thermoplastic processing. This is beneficial to the processing properties of PBAT/TPS composites.

### 3.3. Micromorphological Analysis

**Figure 3:**
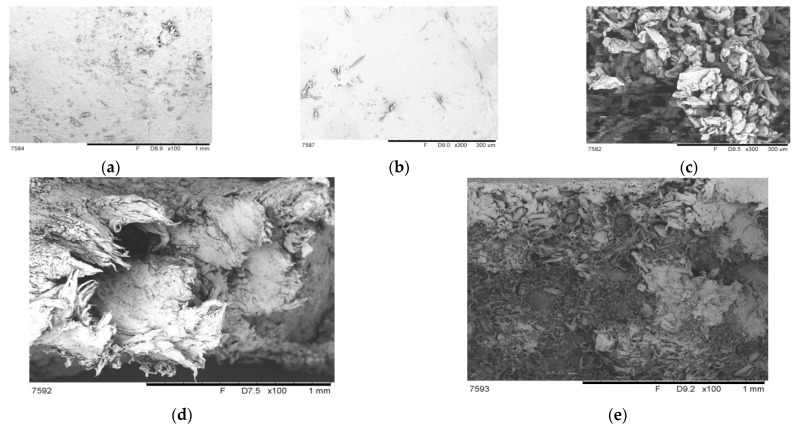
SEM micrographs of the surface of (**a**) TPS, (**b**) PBAT, (**c**) MCC, and the fracture surfaces of the samples with (**d**) 2% MCC and (**e**) 8% MCC.

The surface morphology of TPS, PBAT, and MCC are exhibited in Figure 3a–c, and the fractural surface morphology of the composites with 2% and 8% MCC additions are shown in Figure 3d,e. The surfaces of TPS (Figure 3a) and PBAT (Figure 3b) are nearly smooth. Small particle-like features can be noticed on the TPS surface; possibly, this could be partially crystallized starch due to the crystallization of the aging process. This is a common phenomenon in thermoplastic starch materials and it will limit the mechanical properties such as elongation at break and tensile strength. MCC (Figure 3c) exhibits a rod-like structure with rough surface; the length is estimated to be 50–300 µm. The fractural surface of the sample with 2% MCC (Figure 3d) shows typical ductile characteristics with obvious wire drawings, filaments, and a few holes resulting from the slippage of PBAT molecules. No obvious phase separation and agglomeration of MCC particles are found, indicating the good interfacial adhesion between TPS and PBAT as well as the uniform dispersion of MCC. The improvement in tensile ductility could be attributed to the hydrogen-bond interactions between plenty of hydroxyl groups of MCC and the carbonyl groups of PBAT under a suitable proportion [30]. Furthermore, the roughness of the MCC contributes to good mechanical adhesion as well, when the addition is not in excess [31]. On the contrary, the fractural surface of the sample with 8% MCC (Figure 3e) is comparatively flat. Filamentous features can merely be seen. Instead, a great number of cellular orifices that are filled with rod-like MCC and voids between MCC and the polymer matrix can be found. Excessive MCC agglomerates and causes cavitation—this could be considered to be a defect in the composite [15], and could be adopted to explain the decline in mechanical properties, especially the elongation-at-break decrease from 543% to 78%. Giri et al. [32] also reported a similar condition—that the possible interspace formation led to the drastic reduction in the ductility. Crack initiation and propagation were prone to happen with MCC content over 20% in the PBAT matrix.

### 3.4. FT-IR Analysis

**Figure 4:**
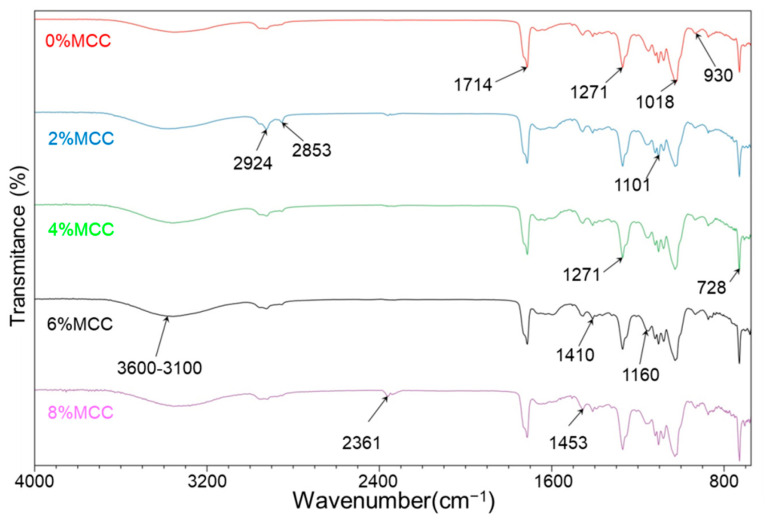
FT-IR spectra of PBAT/TPS/MCC composites.

Figure 4 shows the surface functional group characteristic of the PBAT/TPS/MCC composites. Characteristic bands associated with the –OH stretching vibration, C-O bending vibration, and -OH bending vibration on the pyranose ring of cellulose and starch are found near 3500 cm^−1^, 1080 cm^−1^, and 1101 cm^−1^, respectively. Additionally, the characteristic bands associated with ether C-O-stretching vibration bands are observed in 1160 cm^−1^ and 930 cm^−1^. These absorption bands are the evidence of TPS, MCC, and PBAT in the composites. With the increase in MCC content, the absorption bands near 3500 cm^−1^ are enhanced. This could be explained by the increase in the number of -OH and hydrogen bonds. The change in absorption bands reveals the interaction among starch, PBAT, and MCC. However, the difference in the IR spectrum is generally small, which is similar to the findings of Reis et al. [17]. They concluded that there was no chemical bond breakage and formation, and only a physical entanglement between the three phases existed, except for hydrogen bonds. In addition, there are two absorption bands near 2945 cm^−1^ and 2850 cm^−1^ with respect to the asymmetric- and symmetric-stretching vibrations of aliphatic C-H bonds. The C=O-stretching vibration bands of unsaturated polyester are observed at 1714 cm^−1^, corresponding to the PBAT, which is associated with the in-plane bending vibration of CH_2_ bonds near the 1453 cm^−1^ and 1410 cm^−1^ region. According to the analysis from Ning et al. [33], the bands at 728 cm^−1^ can be contributed to the out-of-plane deformation of the benzene ring on the PBAT.

### 3.5. Thermal Stability Analysis

**Figure 5:**
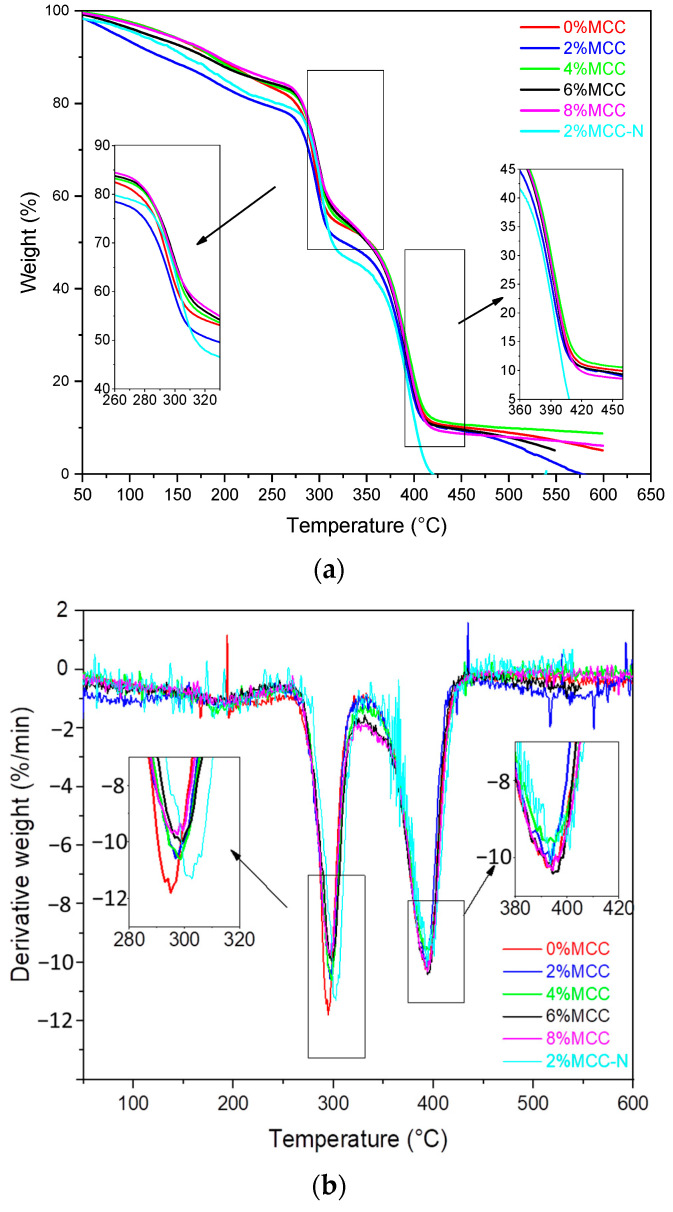
The curves of thermogravimetric analysis (TGA) (**a**) and DTG (**b**).

The thermal stability of the composites is analyzed by thermogravimetry (TGA) and its derivative curves (DTG), as shown in Figure 5a,b. The thermal degradation parameters are listed in Table 3. Dehydration and low-molecule weight compound evaporation occur at 50 °C to 250 °C. This is quite commonly seen in the cellulose and starch composites. Subsequently, the low temperature peak from 270 °C to 320 °C can be attributed to the thermal degradation of TPS. The composites with 2% MCC have the lowest T_onset_ value, which is probably due to the good compatibility between TPS/PBAT/MCC. This promotes heat conduction and thermal degradation. Coincidently, this is consistent with the mechanical result that the composite with 2% MCC has the highest elongation at break. By increasing the MCC content, a closer interaction between MCC, TPS, and PBAT requires a higher level of energy to break down, thus, presenting a stable thermal degradation performance. The degradation step of PBAT exists between the temperatures from 370 °C to 410 °C; the degradation rate between the composites is found to be similar. It may be that MCC is more compatible with hydrophilic TPS compared with hydrophobic PBAT, and it is inclined to form tough structures with TPS under the action of a chain extender. As a result, the curves at the degradation step of PBAT are close and cannot be separated because they are too near. No separate degradation stage of the cellulose can be found in TGA curves, which indicates that cellulose has been completely covered [34]. Generally, the addition of MCC strengthens the thermal stability of the composites, which is represented by the increase in starting temperature (T_onset_), the temperature range of thermal degradation, and the temperature of the maximum thermal degradation rate (T_max_). This could be attributed to MCC promoting the heat transfer in the composites. Importantly, the thermal stability of samples with 4% and 6% MCC are satisfied. Excessive MCC (8%) may inhibit the chain slip because of agglomeration, leading to weaker thermal stabilities. However, its thermal stability is still better than the composites without MCC. Furthermore, the thermal stability decreases without chain extender, according to a lower T_onset_, T_max_ and a higher rate of thermal decomposition. At last, the residues at 550 °C are the fixation of carbon in TPS and PBAT polymers.

### 3.6. DSC Analysis

**Figure 6:**
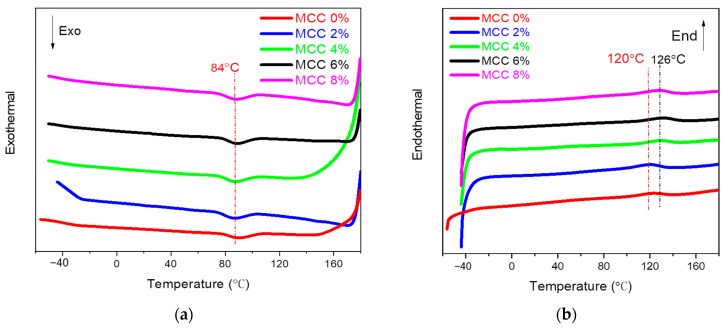
The DSC curves of samples, (**a**) the cooling round and the second heating round (**b**).

The DSC curves of the composites are shown in Figure 6a,b, corresponding to the crystallization transition and melting transition, respectively. Thermal parameters are listed in Table 4. The crystallization transition of the neat PBAT and the pure TPS commonly occur at Ca. 45 °C and Ca. 99 °C [35], whereas the crystallization transition temperature of the composites is obtained at Ca. 87 °C. Most likely, the reason could be the fact that MCC and ADR-4368 enhance the binding capacity of PBAT and TPS, making the two phases more homogeneous in the composites [36]. However, Garalde et al. reported an increase in T_c_ from 83 °C to 90.3 °C without the chain extender [28]. Obviously, the T_c_ and T_m_ of the composites are decreased, and *X_c_* is increased by the addition of the MCC. With 6% MCC, they decreased from 90.3 °C to 88.6 °C and 147.2 °C to 125.7 °C, respectively. Meanwhile, *X_c_* increases from 9.77% to 11.41%, which is similar to that of the PBAT/TPS composites without the chain extender [1]. This can be interpreted as the nucleating effect of the MCC of the PBAT [37]. Contextually, the same effect has been reflected in Figure 6b. With the 6% MCC addition, the endothermic peaks shift to the high temperature, which can also be attributed to the formation of hydrogen bonds between the matrix and MCC. Furthermore, *X_c_* is almost equal to the CI. This demonstrates that the processing of the composite has little effect on the crystalline properties of the composite itself, suggesting that the composite has good processability. The addition of MCC shows less influence on the melting transition of the composites.

### 3.7. Comparison of PABT/TPS Composites with/without the Chain Extender

**Figure 7:**
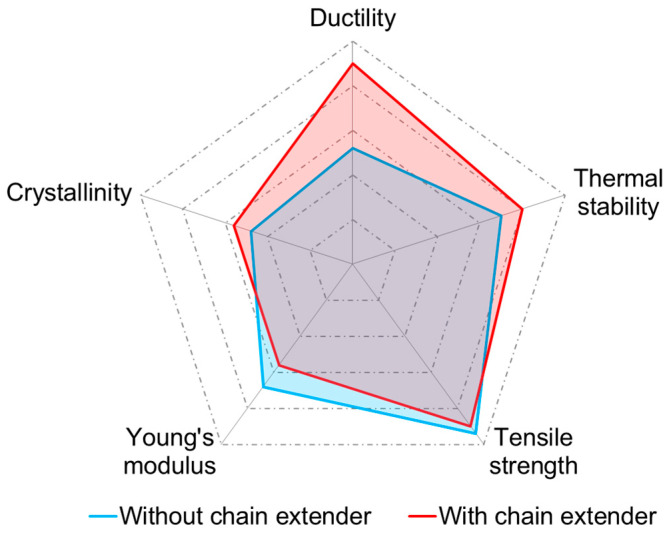
The performance comparison between the no-chain extender and 0.5% chain extender additions.

The performance comparison of the composites with and without the chain extender is shown in Figure 7. These comparisons are based on the same addition of 2% MCC. Both ductility and thermal properties of the composites with the chain extender achieve a remarkable improvement. Notably, the ductility of the composites is nearly two times higher than that without the chain extender. This seems to be slightly lower than the findings of Li et al. [34]. They found that the elongation of the PLA/PBAT composite was improved by ten-fold after the chain extender was added. As we know, there is a contradiction between the tensile strength and ductility. However, tensile strength remains nearly unchanged as ductility improves, which may contribute to the enhancement effect of the chain extender. Furthermore, a lower decomposition rate indicates an improvement in thermal stability by the addition of the chain extender. It also increases the crystallinity of the composites, which might contribute to the enhancement of mechanical properties. As a result, after taking mechanical and thermal properties into consideration, the addition of a chain extender is a potential strategy to strengthen PBAT/TPS composites.

## 4. Conclusions

PBAT/TPS/MCC composite films with chain extender ADR-4368 were fabricated by one-step melt-blending and extrusion. The effect of MCC addition and chain extender was comprehensively evaluated. The results of the mechanical properties showed that the addition of MCC and chain extender enhanced the mechanical properties of the composites to a certain extent, and reached saturation when the addition of MCC reached 6%. Meanwhile, the thermal stability in terms of the melting temperature and thermal degradation temperature were improved with the increase in MCC content (<6%). Although excessive MCC had a certain backlash, most properties were better than those without MCC. Moreover, the results of the XRD analysis, FT-IR analysis, and SEM analysis indicated that PBAT, TPS, and MCC, with the action of a chain extender, formed a new tough interaction, which had good compatibility. The chain extender ADR-4368 improved the ductility, thermal stability, tensile strength, and crystallinity of the composites. Finally, the optimal MCC content was found to be 4%. This study provided a simple strategy to obtain low-cost and high-performance biodegradable films, and it would be helpful to expand the application of PBAT composites in the packaging, mulch field, etc.

## Figures and Tables

**Table 1:** Experimental formula of PBAT/TPS/MCC composites.

Sample Name	PBAT wt%	TPS wt%	MCC wt%	ADR wt%
0% MCC	40	60	0	0.5
2% MCC	40	58	2	0.5
2% MCC-N	40	58	2	0
4% MCC	40	56	4	0.5
6% MCC	40	54	6	0.5
8% MCC	40	52	8	0.5

Note: The amounts of added components are based on dried weight.

**Table 2:** Mechanical properties of PBAT/TPS/MCC composites.

Samples	Tensile Strength (MPa)	Young’s Modulus (MPa)	Elongation at Break (%)
0% MCC	4.03 ± 0.13 ^a^	10.38 ± 0.3 ^a^	556 ± 20 ^a^
2% MCC	4.52 ± 0.22 ^b^	10.18 ± 0.3 ^a^	543 ± 23 ^b^
2% MCC-N	4.58 ± 0.21 ^b^	15.04 ± 0.4 ^b^	303 ± 10 ^c^
4% MCC	5.08 ± 0.36 ^c^	20.58 ± 0.7 ^c^	230 ± 46 ^d^
6% MCC	5.78 ± 0.12 ^d^	30.78 ± 0.6 ^d^	87 ± 45 ^e^
8% MCC	5.64 ± 0.24 ^d^	34.99 ± 1.5 ^d^	78 ± 2 ^e^

Note: Data are the mean of replicate determinations ± standard deviation. ^a,b,c,d,e^ The same letter in the same column indicates no significant difference at the *p* < 0.05 level.

**Table 3:** The parameters of the thermogravimetric analysis of PBAT/TPS/MCC composites.

Samples	Peak	T_onset_ (°C)	Temperature Range (°C)	T_max_ (°C)	Mass Loss at 550 °C (%)
0% MCC	1	283.9	283.9–304.7	295.3	94.8
	2	372.3	372.3–407.8	391.9	
2% MCC	1	282.6	282.6–307.0	297.0	97.5
	2	373.3	373.3–405.5	393.9	
2% MCC-N	1	280.5	280.5–318.9	302.4	99.9
	2	374.2	374.2–408.5	393.6	
4% MCC	1	284.3	284.3–308.0	303.0	91.2
	2	374.4	374.4–410.0	393.9	
6% MCC	1	283.8	283.8–308.8	298.1	94.8
	2	372.9	372.9–406.8	394.0	
8% MCC	1	285.4	285.4–306.7	294.9	93.8
	2	376.4	376.4–408.2	394.4	

**Table 4:** Thermal behaviors of the PBAT/TPS/MCC composites.

Samples	T_c_ (°C)	Δ*H_c_* (J/g)	*X_c_* (%)	*CI* (%)	T_m_ (°C)	Δ*H_m_* (J/g)
0% MCC	90.3	4.456	9.77	9.68	123.0	2.571
2% MCC	86.5	5.133	11.26	11.28	120.2	3.193
4% MCC	87.2	5.373	11.78	11.82	123.2	2.738
6% MCC	88.6	5.183	11.37	11.41	125.7	3.336
8% MCC	88.5	5.354	11.74	11.80	120.6	3.089

## Data Availability

Not applicable.
